# Development of a Predictive Model for Skin-to-Skin Contact Immediately after Birth: A Cross-Sectional Study

**DOI:** 10.3390/children11050577

**Published:** 2024-05-10

**Authors:** María Antonia Díaz-Ogallar, Antonio Hernández-Martínez, Manuel Linares-Abad, Juan Miguel Martínez-Galiano

**Affiliations:** 1 San Agustin Hospital, Andalusian Health Service, 23700 Linares, Spain; mado0003@red.ujaen.es; 2 Nursing Department, University of Jaen, 23071 Jaen, Spain; mlinares@ujaen.es; 3 Department of Nursing, Physiotherapy and Occupational Therapy, Ciudad Real Faculty of Nursing, University of Castilla-La Mancha, 13071 Ciudad Real, Spain; antonio.hmartinez@uclm.es; 4 Consortium for Biomedical Research in Epidemiology and Public Health (CIBERESP), 28029 Madrid, Spain

**Keywords:** predictive model, skin-to-skin contact, newborn, postpartum, mother–child relationship

## Abstract

The aim of this study was to develop and validate a predictive model for the establishment of skin-to-skin contact immediately after birth. A descriptive cross-sectional study was conducted during the last trimester of 2022 and the first trimester of 2023 with women who had given birth in Spain. A questionnaire containing sociodemographic, psychosocial, and health variables referring to the mother and the newborn, as well as the Bond and Attachment questionnaire (VAMF, *for its name in Spanish*) for the analysis of the mother–child bond and attachment, were administered. A multivariate analysis was performed, and areas under the ROC curve (AUC) with their 95% confidence intervals (CI) and the predictive characteristics of these models were estimated. In total, 1077 women participated. The prevalence of early skin-to-skin contact after delivery was 50.2% (468) in the derivation cohort and 49.8% (464) in the validation cohort. Multivariate analysis showed that prematurity, type of delivery, and birth experience were statistically significant, so they were included in the model (*p* ≤ 0.05). The predictive ability (AUC ROC) was good in both the derivation cohort, yielding 0.92 (95% CI: 0.89–0.95), and in the validation cohort, yielding 0.89 (95% CI: 0.85–0.93). This study developed a predictive model identifying factors facilitating early skin-to-skin contact between a mother and her newborn immediately after birth.

## 1. Introduction

Skin-to-skin contact (SSC) is the procedure by which a newborn is placed naked, or occasionally covered with a diaper or hat, in a prone position with their head tilted on their mother’s bare torso immediately after birth, with the exposed area of the infant covered with a blanket or towel [1,2,3,4].

SSC is recommended to be facilitated immediately after birth, or in the first 5–10 min after birth, and to last at least one hour. However, this time varies according to the different protocols and clinical practice guides, extending until the first 120 or 180 min of life of the newborn [2,3,5,6,7,8]. This postnatal period, called the “sensitive period”, was described in the 1970s by Klaus and Kennell [9,10] and is essential in establishing a pattern of reciprocal interaction between mother and child [10]. This period is characterized by the physiological states of both, wherein the mother has high levels of oxytocin and the newborn has very high levels of catecholamines [5]. Full-term newborns activate a series of innate behaviors after birth when placed in SSC with their mothers, including the activation of the olfactory cortex by colostrum during the first day of life [1]. While mother and child engage in uninterrupted SSC during this period, an internal process is activated in the newborn consisting of a series of innate behaviors observable in nine stages: crying at birth, relaxation, awakening, activity, rest, crawling, familiarization, suction, and sleep [5].

SSC provides numerous benefits for both a newborn and their mother. In particular, SSC allows better adaptation of the newborn to extrauterine life [1,11]. The thermoregulation of the infant during SSC is not altered, maintaining temperatures similar to and sometimes even higher than those of newborns who do not receive SSC [1,5,7,11]. For premature newborns, SSC improves cardiorespiratory regulation compared to those who do not receive SSC after birth [1,5,7,11]. SSC is closely related to breastfeeding, increasing the success of the infant’s latching to the breast during the first hour of life, increasing the probability of exclusive breastfeeding during the first months of life and its prolongation over time [1,7,11,12,13,14]. Furthermore, immediate SSC favors the colonization of the microbiome, especially for infants born by cesarean section who do not pass through the birth canal, consequently lacking the colonization they would have acquired were they to pass through it [5]. As for the mother, SSC increases oxytocin levels, promotes uterine contraction, reduces the risk of hemorrhage, shortens the delivery phase of labor, reduces maternal anxiety, and increases the confidence and psychological well-being of the mother [1,5,10,13,15,16]. Furthermore, early and immediate SSC between mother and child after birth has been shown to have a positive impact on the psychological aspects of the mother–child relationship, favoring the formation of a postnatal bond and attachment [1,5,7,9,10,17]. Likewise, it is a factor that reduces the possibility of developing post-traumatic stress disorder related to the experience of childbirth and postpartum depression [18,19].

Few exceptions contraindicate SSC after birth; until relatively recently, among these exceptions was delivery by cesarean section [1,13]. It has been proven that SSC is safe for mothers who have given birth by cesarean section as long as the health status of the mother and/or newborn does not contraindicate it [1,5,11,20]. Among the potential complications that may arise during SSC after birth is sudden and unexpected postnatal collapse, which affects apparently healthy newborns and presents an etiology that is multifactorial. It is believed that it could be caused by respiratory difficulty and failure, and observation of the mother and child during SSC by healthcare personnel is essential, although this complication is not related to the practice of SSC [2,4,5,21].

The practice of SSC after childbirth is a safe practice that provides numerous benefits for both the mother and the newborn in the short and long term. Despite this, the percentage of women who establish SSC immediately after giving birth is highly variable depending on the country, ranging from a prevalence of less than 1% in Tanzania to 98% in Croatia [22]. In Spain, the prevalence is estimated to be around 64.5–69.5%, with an increasing trend of early SSC practice [18,23]. Some maternal and neonatal factors that are associated with early SSC have been identified, such as the type of delivery, low weight or prematurity of the newborn, or maternal beliefs about SSC, although they are associated with contradictory results [11,15,24,25,26]. Therefore, developing a predictive model that can help to determine the probability of SSC immediately after birth is essential for clinical practice, allowing health personnel to implement and reinforce measures that promote SSC after birth. In addition, it would be useful to know what factors are related to this practice, as this would allow those factors to be reinforced and ensure their success and acceptance by both the mother and the child, in addition to the health professionals involved in the birth and immediate postpartum process.

The present study aims to develop and validate a predictive model for facilitating SSC immediately after delivery.

## 2. Methods

### 2.1. Study Design and Subject Selection

This cross-sectional study was conducted during the last trimester of 2022 and the first trimester of 2023 with women who had given birth in Spain.

To estimate the sample size, the maximum modeling criterion was followed, where for each independent variable included in the model, 10 subjects who present the problem under study must be included [27]. That is, for each independent variable, 10 maternal-child dyads for whom SCC was facilitated immediately after childbirth are required. Considering that around 65% of women will engage in SSC, a minimum of 200 women who facilitated SSC and 108 women who did not are required to include 20 predictor variables in the initial model (total = 308 women). However, it was decided to include the largest number of women to improve the statistical power of the estimates and to distribute half of the dyads randomly to form a derivation cohort and a model validation cohort.

The established inclusion criteria were women whose age was between 18 and 45 and who had given birth in the last 18 months and provided informed consent for participation in this study. The exclusion criteria were women who had a multiple birth (two or more newborns) and who did not speak or know the Spanish language (language barrier).

To recruit women for our study sample, different associations related to pregnancy, childbirth, and postpartum support groups for breastfeeding and parenting throughout the Spanish territory were contacted. After applying the inclusion and exclusion criteria, the participants were informed about the objective and mode of participation of this study, and after providing informed consent for participation in this research, they were administered the questionnaire.

### 2.2. Data Collection

To collect data, a questionnaire containing sociodemographic, psychosocial, and health variables related to the mother and the newborn was administered.

Dependent variable: Skin-to-skin contact immediately after birth.

Independent variables:

1. Mother: age of the mother, marital status, income level, current affliction with illness, number of pregnancies, type of birth, number of children, pregnancy with high obstetric risk, planned pregnancy, use of assisted reproduction techniques, maternal education during pregnancy, affliction with health problems during pregnancy, affliction with anxiety during pregnancy, childbirth and postpartum ailments, affliction with mental health problems, affliction with depression before or during pregnancy, perceived support from the partner and the family, whether the mother was a smoker, and the birth experience.
2. Newborn: age (months), birth weight, and prematurity.

The mother–child bond and attachment were measured with the VAMF questionnaire developed by Diaz-Ogallar et al. in 2024 [28]. This questionnaire comprises a total of 29 items divided into two subscales, one for bond (VAMF-bond), consisting of 16 items, and another for attachment (VAMF-attachment), with 13 items. The VAMF questionnaire has good psychometric capabilities, with an internal consistency of α = 0.836. Each item has 4 response options, namely, never, sometimes, often, and always, with a score ranging from 1 to 4. The higher the score, the higher the quality of the bond and attachment between mother and child. The dyad is considered to reflect impaired bonding or attachment if its total score on the questionnaire is below the 10th percentile.

### 2.3. Statistical Analysis

The statistical analysis was conducted using the program SPSS 28.0. First, descriptive statistical analyses were performed, using means with standard deviations (SDs) for continuous variables and absolute and relative frequencies for categorical variables.

Next, a bivariate analysis of the predictive factors was performed using the Student’s *t* test for quantitative variables (mother’s age) and the Chi-squared test for qualitative variables (the rest of the variables). These predictive factors were previously identified in the literature as factors related to establishing SSC immediately after birth. They were subsequently included in a multivariate model using stepwise backward elimination (RV in SPSS).

Statistical reliability parameters for the model were analyzed, including -2LL, Cox–Snell R2, and Nagelkerke R2, accounting for sensitivity, specificity, positive and negative predictive values, and positive likelihood ratio for the different probabilities of the model, both for the derivation cohort and for the validation cohort.

In the next step, the area under the curve (AUC) of the receiver operating characteristic (ROC) with their 95% confidence intervals (CI) for the validation cohort was calculated for the predictive model. To qualitatively analyze the prediction, the Swets criterion was used considering the following ranges: 0.5–0.6 (very poor), 0.6–0.7 (poor), 0.7–0.8 (satisfactory), 0.8–0.9 (good), and 0.9–1.0 (excellent) [29].

Finally, the derivation and validation cohorts were compared using the Chi-squared and Student’s *t*-tests to calculate the qualitative and quantitative variables, respectively.

## 3. Results

A total of 1077 women participated in this study; 544 were part of the derivation cohort, and 533 were part of the validation cohort. The mean age of the participants in this cohort was 34.7 years; 60.3% (328) were married, and 90.4% (492) did not have any illness at the time of the study. A total of 50.9% (277) were primiparous, 40.6% (221) had some kind of health problem during pregnancy, and 14.3% (78) had a high-obstetric-risk pregnancy. In total, 60.8% (331) had normal births, and 68.4% (372) defined their birth experience as good or very good, 1.5% (8) of the newborns were premature (Table 1).

The prevalence of SCC after birth was 50.2% (468) in the derivation cohort and 49.8% (464) in the validation cohort.

The statistically significant variables (*p* ≤ 0.05) in the bivariate analysis for SSC were the following: income level, number of pregnancies, number of vaginal births, having had a previous cesarean section, number of children, use of assisted reproductive techniques, having been diagnosed with depression before/during pregnancy, support received from the partner, the type of delivery, and the experience of delivery (Table 2).

Finally, the multivariate analysis showed that the variables prematurity, type of birth, and birth experience were statistically significant, so they were included in the model (*p* ≤ 0.05). Newborn prematurity (aOR: 0.10; 95% CI: 0.01–0.94), instrumental birth (aOR: 0.31; 95% CI: 0.10–0.96), elective cesarean section (aOR: 0.05; 95% CI: 0.01–0.15), and emergency cesarean section (aOR: 0.03; 95% CI: 0.01–0.07) reduce the probability of SCC following birth. On the other hand, a birth experience perceived as average (aOR: 2.53; CI: 1.14–5.61) or good or excellent (aOR: 8.19; CI: 3.40–19.70) increases the probability of SCC immediately after birth compared to that for mothers who had a bad experience (Table 3).

**Figure 1:**
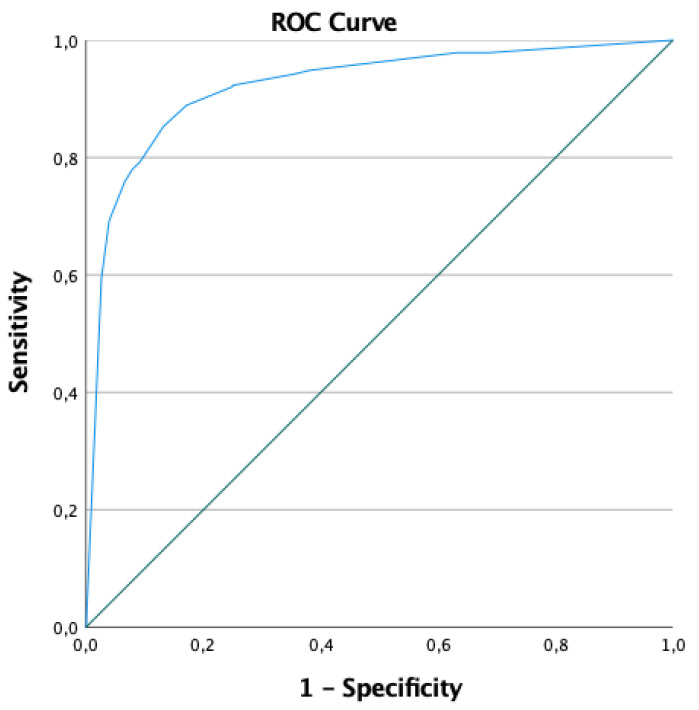
ROC curve of the predictive model regarding the derivation cohort.

**Figure 2:**
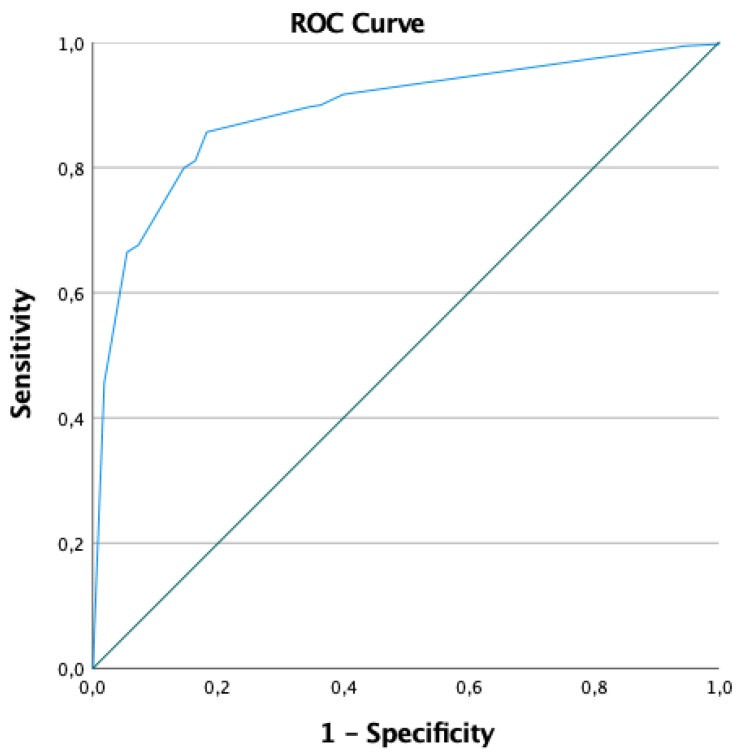
ROC curve of the predictive model regarding the validation cohort.

The predictive ability of this model was good for both the derivation and validation cohorts. The AUC of the ROC was 0.92 (95% CI: 0.89–0.95) for the derivation cohort (Figure 1) and 0.89 (95% CI: 0.85–0.93) for the validation cohort (Figure 2). The predictive characteristics of the model for the derivation and validation cohorts were also considered for different probabilities (Table 4).

Finally, the variables for both cohorts were compared, revealing no statistically significant differences except for the current illness variable (*p* = 0.027) (Table 5).

## 4. Discussion

A predictive model was developed to determine the probability of mother–child SSC immediately after delivery. This model presented a good predictive capacity in the derivation and validation cohorts, finding as predictive factors for SSC the prematurity of the newborn, the type of birth, and the maternal assessment of the birth experience.

One of the factors found in this model that influences the establishment of SSC is the prematurity of the newborn. The World Health Organization (WHO) recommends the establishment of SSC with premature newborns as soon as they are clinically stable [30]. In line with our results, different studies associated prematurity with a lower probability of establishing SSC due to different factors. Thus, in their study, Lee et al. [31] identified the factors that hindered the practice of SSC with premature newborns, one of them being the difference found regarding the definition of “clinical stability” between institutions and professionals. Another of the barriers identified for SSC constitutes the types of equipment and devices used for the care of premature newborns, such as the presence of catheters, endotracheal tubes, mechanical ventilation, etc. [31].

The type of birth is another factor that has been found to influence the establishment of early SSC between mother and child. The WHO [32] suggests that cesarean birth rates should be lower than 10–15%, while in Spain, the cesarean birth rate is much higher, around 25.7% [33,34]. The Spanish Association of Pediatrics (AEPED) indicates that skin-to-skin contact in cesarean delivery could be hindered by the reluctance of the professionals to facilitate it, finding as drawbacks the compromise of the sterility of the operating room in facilitating SSC, the hypothermia that the newborn may suffer due to the environmental conditions that the operating room must have, the underlying complications of the surgery, and the monitoring of the newborn during SSC and transfer to the Post-Surgical Resuscitation Unit, in addition to which professional is in charge of it not being defined and the lack of training of staff in this area [33,34]. The WHO [35] recommends the initiation of skin-to-skin contact immediately after a cesarean section is performed with epidural analgesia or when the mother is alert enough to hold her child in the case of a cesarean section performed with general anesthesia. Despite these recommendations, in line with our results, several studies identify cesarean delivery as a factor for the mother not establishing SSC with her newborn. In their research, Döblin et al. [36] reported that mothers who had an instrumental or cesarean birth had a worse birth experiences than those who had a normal vaginal birth. Koopman et al. [24] found that among the factors that hinder the implementation of early SSC in gynecology and obstetrics units and NICUs are institutional factors, such as the skills of professionals or the presence of catheters or electrodes that hinder the practice of SSC, as well as the training of professionals and traditional clinical practices. These difficulties are greater in the case of cesarean births, where the need for more health personnel, the size of the operating room, and the possible discomfort of the mother are additional barriers [24]. All these factors may influence the reduction in the facilitation of SCC between the mother and the newborn when the latter is born by cesarean section, as has been identified in our study.

Another of the factors found to influence the establishment of SSC is the mother’s subjective assessment of the birth experience (wherein a woman has experienced a situation during childbirth that she perceives to be not appropriate, either due to the treatment received or because clinical practices are carried out that have not been duly explained or justified). In line with the results found in the present study, Suárez-Cortés et al. [37] reported that women whose birth plans had been agreed to by their midwives and ultimately carried out had higher satisfaction in this process and described their experience as being positive, finding better results in the practice of SSC in comparison with women who did not have a consensual birth plan, as found by Fernandez-Turienzo et al. [38] in their study. Likewise, another study that related the treatment or experience of a woman during childbirth to early SSC stated that women who undergo early SSC are less likely to be subject to inadequate treatment during childbirth, so it seems clear that there is a relationship between the experience of a woman during childbirth and the early establishment of SSC [39].

Among the limitations of the present study, it is worth highlighting that this analysis was carried out on the Spanish population, so it would need to be validated in other populations. As it is a questionnaire, there is the possibility that it is affected by selection bias associated with the non-response of the participants. However, there are no indications or reasons to think that the women who did not answer would have responded differently from those who did. Another possible bias in this study is memory bias due to the fact that some information is based on maternal recall. A priori, this is very relevant information that a woman can hardly forget.

In addition to confirming the influence of different factors that were already well known for the establishment of early skin-to-skin contact between a mother and her newborn, the experience of the treatment received by the mother during delivery assistance emerged from the results. For this reason, future research along these lines should be conducted in order to promote the adequate treatment of women during childbirth with the aim of promoting early skin-to-skin contact between a mother and her newborn and the benefits that this entails for the health of the child.

## 5. Conclusions

A predictive model that allows us to identify the factors that favor the practice of SSC between mother and child immediately after birth, namely, the prematurity of the newborn, the type of birth, and the maternal assessment of the birth experience, was developed. Knowing the factors that favor the practice of SSC opens the door for health professionals in contact with dyads during the birth process to reevaluate the procedures carried out and develop new strategies to empower mothers and reinforce this practice, which is beneficial for establishing the relationship between mother and child.

## Figures and Tables

**Table 1:** Sociodemographic and clinical characteristics of the study sample.

Variable	n (%) N = 544	Mean (SD)
**Age mother (years)**		34.7 (3.97)
**Civil status**		
Married	328 (60.3)	
Common-law couple	88 (16.2)	
Single	123 (22.6)	
Divorced	4 (0.7)	
Widowed	1 (0.2)	
**Income level**		
<1000 euros/month	98 (18.0)	
Between EUR 1000 and 1999 per month	292 (53.7)	
Between EUR 2000 and 2999 per month	126 (23.2)	
>EUR 3000/month	28 (5.1)	
**Current illness**		
No	492 (90.4)	
Yes	52 (9.6)	
**No. of pregnancies**		
One	277 (50.9)	
Two	173 (31.8)	
Three or more	94 (17.3)	
**Vaginal births**		
None	106 (19.5)	
One	312 (57.4)	
Two or more	126 (23.1)	
**Cesarean sections**		
No	414 (76.1)	
Yes	130 (23.9)	
**No. of children**		
One	380 (69.9)	
Two	147 (27.0)	
Three or more	17 (3.1)	
**High-risk pregnancy**		
No	466 (85.7)	
Yes	78 (14.3)	
**Planned pregnancy**		
No	52 (9.6)	
Yes	492 (90.4)	
**Fertility treatment**		
No	471 (86.6)	
Yes	73 (13.4)	
**Prenatal education**		
No	162 (29.8)	
Yes	382 (70.2)	
**Health problem during pregnancy**		
No	323 (59.4)	
Yes	221 (40.6)	
**Anxiety during pregnancy, birth, postpartum**		
No	279 (51.3)	
Yes	265 (48.7)	
**Mental health problems**		
No	419 (77.0)	
Yes	125 (23.0)	
**Depression before/during pregnancy**		
No	482 (88.6)	
Yes	62 (11.4)	
**Support received from partner**		
Very low/low	33 (6.1)	
Moderate	94 (17.3)	
High/Very high	417 (76.6)	
**Support received from family**		
Very low/low	57 (10.4)	
Moderate	114 (21.0)	
High/Very high	373 (68.6)	
**Smoker**		
No	487 (89.5)	
Yes	57 (10.5)	
**Prematurity**		
No	533 (98.0)	
Yes	8 (1.5)	
Unknown	3 (0.5)	
**Type of delivery**		
Normal	331 (60.8)	
Instrumental	100 (18.5)	
Planned cesarean section	29 (5.3)	
Emergency cesarean section	84 (15.4)	
**Birth experience**		
Very bad/bad	70 (12.9)	
Ok	102 (18.7)	
Good/Very good	372 (68.4)	
**Skin-to-skin**		
No	76 (14.0)	
Yes	468 (86.0)	

SD: standard deviation.

**Table 2:** Bivariate analysis of the derivation cohort.

	Skin-to-Skin		Bivariate Analysis	
Variable	No n (%) (N = 76)	Yes n (%) (N = 468)	OR 95% CI	*p*-Value
**Age of mother (years)**	34.83 (4.09)	34.63 (3.96)	0.99 (0.93–1.05)	0.691
**Civil status**				0.586
Married	40 (12.2)	288 (87.8)	1 (ref.)	
Common-law couple	13 (14.8)	75 (85.2)	0.80 (0.41–1.57)	0.520
Single	22 (17.9)	101 (82.1)	0.64 (0.36–1.13)	0.120
Divorced	1 (25.0)	3 (75.0)	0.42 (0.04–4.10)	0.453
Widowed	0 (0.0)	1 (100.0)		1.000
**Income level**				**0.039**
<EUR 1000/month	18 (18.4)	80 (81.6)	1 (ref.)	
Between EUR 1000 and 1999/month	47 (16.1)	245 (83.9)	1.17 (0.64–2.14)	0.602
Between EUR 2000 and 2999/month	8 (6.3)	118 (93.7)	**3.32 (1.38–8.00)**	**0.008**
>EUR 3000/month	3 (10.7)	25 (89.3)	1.88 (0.51–6.90)	0.344
**Current illness**				0.467
No	67 (13.6)	425 (86.4)	1 (ref.)	
Yes	9 (17.3)	43 (82.7)	0.75 (0.35–1.62)	
**No. of pregnancies**				0.056
One	46 (16.6)	231 (83.4)	1 (ref.)	
Two	15 (8.7)	158 (91.3)	**2.10 (1.13–3.89)**	**0.019**
Three or more	15 (16.0)	79 (84.0)	1.05 (0.56–1.98)	0.883
**Previous vaginal births**				**<0.001**
None	55 (51.9)	51 (48.1)	1 (ref.)	
One	20 (6.4)	292 (93.6)	**15.75 (8.71–28.46)**	**<0.001**
Two or more	1 (0.8)	125 (99.2)	**134.80 (18.17–1000.40)**	**<0.001**
**Previous cesarean sections**				**<0.001**
No	16 (3.9)	398 (96.1)	1 (ref.)	
Yes	60 (46.2)	70 (53.8)	**0.05 (0.03–0.09)**	
**No of children**				0.104
One	61 (16.1)	319 (83.9)	1 (ref.)	
Two	13 (8.8)	134 (91.2)	**1.97 (1.05–3.71)**	**0.035**
Three or more	2 (11.8)	15 (88.2)	1.43 (0.32–6.43)	0.638
**High-risk pregnancy**				0.075
No	60 (12.9)	406 (87.1)	1 (ref.)	
Yes	16 (20.5)	62 (79.5)	0.57 (0.31–1.06)	
**Planned pregnancy**				0.467
No	9 (17.3)	43 (82.7)	1 (ref.)	
Yes	67 (13.6)	425 (86.4)	1.33 (0.62–2.85)	
**Fertility treatment**				**0.038**
No	60 (12.7)	411 (87.3)	1 (ref.)	
Yes	16 (21.9)	57 (78.1)	**0.52 (0.28–0.96)**	
**Prenatal education**				0.522
No	25 (15.4)	137 (84.6)	1 (ref.)	
Yes	51 (13.4)	331 (86.6)	1.18 (0.71–1.99)	
**Health problem during pregnancy**				0.975
No	45 (13.9)	278 (86.1)	1 (ref.)	
Yes	31 (14.0)	190 (86.0)	0.99 (0.61–1.63)	
**Depression before/during pregnancy**				**0.024**
No	64 (13.3)	418 (86.7)	1 (ref.)	
Yes	12 (19.4)	50 (80.6)	**0.55 (0.33–0.93)**	
**Support received from partner**				0.085
Very low/low	9 (27.3)	24 (72.7)	1 (ref.)	
Moderate	13 (13.8)	81 (86.2)	2.34 (0.89–6.13)	0.085
High/Very high	54 (12.9)	363 (87.1)	**2.52 (1.11–5.71)**	**0.027**
**Support received from family**				0.096
Very low/low	8 (14.0)	49 (86.0)	1 (ref.)	
Moderate	23 (20.2)	91 (79.8)	0.65 (0.27–1.55)	0.328
High/Very high	45 (12.1)	328 (87.9)	1.19 (0.53–2.67)	0.674
**Smoker**				
No	69 (14.2)	418 (85.8)	1 (ref.)	
Yes	7 (12.3)	50 (87.7)		
**Prematurity**				0.904
No	75 (14.1)	458 (85.9)	1 (ref.)	
Yes	1 (12.5)	7 (87.5)	1.14 (0.14–9.39)	
Don’t know	0 (0.0)	3 (100.0)		
**Type of delivery**				**<0.001**
Normal	6 (1.8)	325 (98.2)	1 (ref.)	
Instrumental	10 (10.0)	90 (90.0)	**0.17 (0.06–0.47)**	**0.001**
Planned cesarean section	9 (31.0)	20 (69.0)	**0.04 (0.01–0.13)**	**<0.001**
Emergency cesarean section	51 (60.7)	33 (39.3)	**0.01 (0.01–0.03)**	**<0.001**
**Birth experience**				**<0.001**
Very bad/bad	35 (50.0)	35 (50.0)	1 (ref.)	
Neither good nor bad	26 (25.5)	76 (74.5)	**2.92 (1.53–5.58)**	**0.001**
Good/Very good	15 (4.0)	357 (96.0)	**23.80 (11.85–47.80)**	**<0.001**

Bold: Statistically significant differences. CI: confidence intervals. OR: Odds ratio. ref.: reference.

**Table 3:** Predictive model of skin-to-skin contact.

Number of Events in the Derivation Cohort	468 (86.0%)		
Number of Events in the Validation Cohort	464 (87.1%)		
Risk Factor	Coeff *	Odds Ratio (95% CI)	*p*-Value
**Type of delivery**			**<0.001**
Normal		1 (ref.)	
Instrumental	−1.159	**0.31 (0.10–0.96)**	**0.043**
Planned cesarean section	−3.102	**0.05 (0.01–0.15)**	**<0.001**
Emergency cesarean section	−3.641	**0.03 (0.01–0.07)**	**<0.001**
**Birth experience**			**<0.001**
Very bad/bad		1 (ref.)	
Ok	0.927	**2.53 (1.14–5.61)**	**0.023**
Good/Very good	2.103	**8.19 (3.40–19.70)**	**<0.001**
**Prematurity**			**0.044**
No		1 (ref.)	
Yes	−2.323	**0.10 (0.01–0.94)**	
**Constant**	2.371		
**AUC ROC Derivation Cohort**		**0.92 (0.89–0.95)**	**<0.001**
	2LL: 248.252 Cox-Snell R^2^: 0.297 Nagelkerke R^2^: 0.536		
**AUC ROC Validation Cohort**		**0.89 (0.85–0.93)**	**<0.001**

Bold: Statistically significant differences. CI: confidence intervals. Coeff *: coefficient. OR: Odds ratio. ref.: reference.

**Table 4:** Predictive characteristics of the model for different probabilities.

Probability	Sensitivity	Specificity	PPV	NPV	LR+	LR−
**Higher 0.5**						
Derivation Cohort	94.9	61.8	93.9	66.2	2.48	0.08
Validation Cohort	95.3	60.9	94.2	65.6	2.44	0.08
**Higher 0.6**						
Derivation Cohort	94.2	64.5	94.2	64.5	2.65	0.09
Validation Cohort	94.0	65.2	94.8	61.6	2.70	0.09
**Higher 0.7**						
Derivation Cohort	92.3	75.0	95.8	61.3	3.69	0.10
Validation Cohort	90.1	73.9	95.9	52.6	3.45	0.13
**Higher 0.8**						
Derivation Cohort	85.3	86.8	97.6	48.9	6.46	0.17
Validation Cohort	84.5	84.1	97.3	44.6	5.31	0.18
**Higher 0.9**						
Derivation Cohort	78.0	92.1	98.4	40.5	9.87	0.24
Validation Cohort	77.6	84.1	97.3	44.6	4.88	0.27

PPV: Positive Predictive Value; NPV: Negative Predictive Value; LR+: Positive Likelihood Ratio; LR−: Negative Likelihood Ratio. This table presents the predictive characteristics for different cut-off points of probabilities estimated with the created model. These cut-off points were >0.5, >0.6, >0.7, >0.8, and >0.9.

**Table 5:** Comparison of variables in the derivation and validation cohorts.

	Cohorts		
Variable	Derivation n (%) (N = 544)	Validation n (%) (N = 533)	*p*-Value
**Age mother (years) Mean (SD)**	34.7 (3.97)	34.6 (3.89)	0.855
**Civil status**			0.420
Married	328 (52.1)	301 (47.9)	
Common-law couple	88 (49.4)	90 (50.6)	
Single	123 (47.3)	137 (52.7)	
Divorced	4 (57.1)	3 (42.9)	
Separated	0 (0.0)	2 (100.0)	
Widowed	1 (100)	0 (0.0)	
**Income level**			0.686
<EUR 1000/month	98 (46.9)	111 (53.1)	
Between EUR 1000 and 1999/month	292 (51.0)	281 (49.0)	
Between EUR 2000 and 2999/month	126 (52.3)	115 (47.7)	
>EUR 3000/month	28 (51.9)	26 (48.1)	
**Current illness**			**0.027**
No	492 (51.7)	459 (48.3)	
Yes	52 (41.3)	74 (58.7)	
**No. of pregnancies**			0.076
One	277 (47.4)	307 (52.6)	
Two	173 (53.2)	152 (46.8)	
Three or more	94 (56.0)	74 (44.0)	
**Vaginal births**			0.384
None	106 (49.3)	109 (50.7)	
One	312 (49.4)	319 (50.6)	
Two or more	126 (54.5)	105 (45.5)	
**Cesarean sections**			0.370
No	414 (51.3)	393 (48.7)	
Yes	130 (48.1)	140 (51.9)	
**No. of children**			0.318
One	380 (49.3)	391 (50.7)	
Two	147 (54.4)	123 (45.6)	
Three or more	17 (47.2)	19 (52.8)	
**High-risk pregnancy**			0.822
No	466 (50.7)	454 (49.3)	
Yes	78 (49.7)	79 (50.3)	
**Planned pregnancy**			0.754
No	52 (52.0)	48 (48.0)	
Yes	492 (50.4)	485 (49.6)	
**Fertility treatment**			0.748
No	471 (50.3)	465 (49.7)	
Yes	73 (51.8)	68 (48.2)	
**Prenatal education**			0.854
No	162 (50.9)	156 (49.1)	
Yes	382 (50.3)	377 (49.7)	
**Health problem during pregnancy**			0.825
No	323 (50.2)	320 (49.8)	
Yes	221 (50.9)	213 (49.1)	
**Anxiety during pregnancy, birth, postpartum**			0.788
No	279 (50.9)	269 (49.1)	
Yes	265 (50.1)	264 (49.9)	
**Mental health problems**			0.164
No	419 (51.7)	391 (48.3)	
Yes	125 (46.8)	142 (53.2)	
**Depression before/during pregnancy**			0.234
No	482 (49.9)	484 (50.1)	
Yes	62 (55.9)	49 (44.1)	
**Support received from partner**			0.877
Very low/low	33 (50.8)	32 (49.2)	
Moderate	94 (52.2)	86 (47.8)	
High/Very high	417 (50.1)	415 (49.9)	
**Support received from family**			0.383
Very low/low	57 (55.9)	45 (44.1)	
Moderate	114 (52.3)	104 (47.7)	
High/Very high	373 (49.3)	384 (50.7)	
**Smoker**			0.173
No	487 (49.8)	490 (50.2)	
Yes	57 (57.0)	43 (43.0)	
**Prematurity**			0.147
No	533 (50.8)	516 (49.2)	
Yes	8 (33.3)	16 (66.7)	
Unknown	3 (75.0)	1 (25.0)	
**Type of delivery**			0.734
Normal	331 (50.8)	321 (49.2)	
Instrumental	100 (50.8)	97 (49.2)	
Planned cesarean section	29 (43.9)	37 (56.1)	
Emergency cesarean section	84 (51.9)	78 (48.1)	
**Birth experience**			0.191
Very bad/bad	70 (57.9)	51 (42.1)	
Ok	102 (51.5)	96 (48.5)	
Good/Very good	372 (49.1)	386 (50.9)	
**Skin-to-skin**			0.884
No	75 (52.4)	68 (47.6)	
Yes	468 (50.2)	464 (49.8)	
Unknown	1 (50.0)	1 (50.0)	

Bold: Statistically significant differences.

## Data Availability

The data presented in this study are available on request from the corresponding author. The data are not publicly available due to restrictions related to privacy.
